# Influences of Adhesion Variability on the “Living” Dynamics of Filamentous Bacteria in Microfluidic Channels

**DOI:** 10.3390/molecules21080985

**Published:** 2016-07-28

**Authors:** Justin P. Jahnke, Jessica L. Terrell, Austin M. Smith, Xuanhong Cheng, Dimitra N. Stratis-Cullum

**Affiliations:** 1 U.S. Army Research Laboratory, Adelphi, MD 20783, USA; justin.jahnke2.ctr@mail.mil (J.P.J.); jessica.l.terrell7.ctr@mail.mil (J.L.T.); austin.smith2@utsa.edu (A.M.S.); 2 Department of Materials Science and Engineering, Bioengineering Program, Lehigh University, Bethlehem, PA 18015, USA

**Keywords:** filamentous bacteria, fimbriae, mannosylated substrate, adhesion, orientation, microfluidic, buckle, shear flow

## Abstract

Microfabricated devices have increasingly incorporated bacterial cells for microscale studies and exploiting cell-based functions in situ. However, the role of surface interactions in controlling the bacterial cell behavior is not well understood. In this study, microfluidic substrates of varied bacterial-binding affinity were used to probe the interaction-driven behavior of filamentous *Escherichia coli.* In particular, cell alignment under controlled shear flow as well as subsequent orientation and filamentation were compared between cells presenting distinct outer membrane phenotypes. We demonstrated that filaments retained position under flow, which allowed for dynamic single-cell monitoring with in situ elongation of over 100 μm for adherent cells. This maximum was not reached by planktonic cells and was, therefore, adhesion-dependent. The bound filaments initially aligned with flow under a range of flow rates and their continual elongation was traced in terms of length and growth path; analysis demonstrated that fimbriae-mediated adhesion increased growth rate, increased terminal length, as well as dramatically changed the adherent geometry, particularly buckling behavior. The effects to filament length and buckling were further exaggerated by the strongest, specificity-driven adhesion tested. Such surface-guided control of the elongation process may be valuable to yield interesting “living” filamentous structures in microdevices. In addition, this work may offer a biomedically relevant platform for further elucidation of filamentation as an immune-resistant morphology. Overall, this work should inspire broader exploration of microfabricated devices for the study and application of single bacterial cells.

## 1. Introduction

With a growing interest in using whole bacterial cells as living components in fuel cells, biosensors, and bioactuators [1,2,3,4], there is a demand of critical understanding and control of bacterial interaction with engineered substrates. Knowledge of such interactions is also important in the biomedical field and food industry, given the central role of adhesion in pathogenicity and biofilm formation. At the same time, bacterium-substrate interactions often take place in the presence of flowing liquid, from shear flow in microdevices and physiological conditions to turbulent flow in natural water resources and manmade macrohabitats. The analysis of bacterial behavior on a surface without flow may not reflect the natural state of cells [5,6]. Elucidating bacterium-substrate interaction under a controlled flow, thus, has implications in many contexts from developing hybrid devices with whole cells to treating infections and controlling biofilms [7,8,9].

Bacteria have several modes of colonization, with fimbriae surface organelles allowing for stable attachment under flow conditions [10]. Type 1 fimbriae are long rod-like assemblies of proteins that extend from the cellular outer membrane (in Gram-negative bacteria) with prevalence of 200–500 copies per cell. Structurally, fimbriae reach up to 1 μm in length and are approximately 7–10 nm in diameter [11]. Nonspecific cellular adhesion is mediated by hydrophobic interactions between the fimbriae and surface [12]. Further, the tip protein of Type 1 fimbriae bind specifically to glycosylated surfaces (i.e., mammalian tissues) with high mannose content [13]. Single mannose residues facilitate a binding state that is enhanced by tensile force (e.g., shear force of fluid flow) applied to the cell [14]. Additionally, fimbriae attach to oligomeric mannosylated surfaces (found on uroepithelial cells) with a greater binding strength than to single mannose residues [15].

While bacteria normally grow in highly-regulated shapes [16,17], bacteria can also grow as very long cells (10 s to 100 s of microns), termed filaments. Filamentation is a process by which cell division is inhibited despite continued cell proliferation, resulting in elongated, multi-nucleated cells. This occurs by inhibiting the midcell formation of a septal ring, which serves to contract and pinch off two daughter cells [18]. In *Escherichia coli*, this may occur as an adaptive response to DNA damage, high pressures [19], or temperature-induced stress [20,21,22]. The tendency for mutagenic DNA repair during filamentation may favor the development of antibiotic resistance [23]. Filamentation is also a mechanism of persistence to promote host attachment and evade engulfment by immune cells [5,24,25,26]. Additionally, certain antibiotics trigger filamentation by interfering with chromosomal replication, such as with ciprofloxacin [27], or through inhibition of structural peptidoglycan synthesis by β-lactams. In particular, aztreonam, a β-lactam, inhibits cell division by directly inactivating FtsI, a transpeptidase required for septal peptidoglycan synthesis. The cells are otherwise structurally- and chromosomally-intact, such that cell proliferation may continue for multiple generations asymptomatically, although the accumulation of structural defects renders filaments prone to lysis [28,29]. Nevertheless, bacterial behavior, such as swimming [30] and chemotaxis [31], are retained, and bacteria can redivide if the source of stress is removed [32]. 

In addition to the interest in fundamental bacteriology, there has also been interest in developing bacterial filaments for biotechnological applications, due to the high aspect ratio and large size relative to normal bacterial cells. Filaments can be induced by overexpressing cell wall proteins [33], and these filaments have been used as a template for polyelectrolytes [34]. It has also been shown that filaments grown in constrained environments can be forced to adopt curved shapes, such as corkscrews, that are retained once the constraint is removed [35]. Interestingly, in microfluidic devices, it has been shown that shear can be used to align growing bacteria filaments on surfaces. In this case, the filaments were aligned with shear to span a passivated region between two adhesive patches [36]. Despite extensive studies of filamentous cells on substrates and preliminary observation of filamentation differences between adherent and planktonic states, the role of surface interactions on the growth and terminal length of the bacterial filaments is poorly understood [37,38].

Given the interest in better understanding the interaction of filamentous bacteria with surfaces under shear, this study analyzed the influence of substrate affinity on filamentation dynamics in a microfluidic device. Microfluidic technology has grown to be a powerful tool in microbiology [39]. The capability to create single cell arrays [40] and precisely control the physical and chemical micro-environment around individual cells [41] makes it possible to analyze individual bacterial cells on the population, cellular [42], and molecular levels [40]. In addition, microfluidic devices are readily engineered to generate spatially-defined, versatile flow profiles, enabling the study of flow influence on microorganism metabolism [43,44], transport [45,46], planktonic cell locomotion [47,48], and dynamic biofilm formation [49,50,51,52]. On the dimension of a single bacterium, the flow field is present as a linear velocity profile, or simple shear, which can be readily engineered in microfluidic devices. Here, microfluidic devices were used to create a controlled shear environment around single cells. Different levels of mannosylation were introduced to the microfluidic substrates to control cell-device interactions. Filamentous cells with different fimbriae densities were studied in the microchannel. Through real-time imaging of single cell morphology, distinctive cell behaviors have been observed and discussed as a function of adhesion strength and flow conditions. 

## 2. Results

### 2.1. Initial Alignment of Adhered Filamentous Bacteria by Shear 

**Figure 1:**
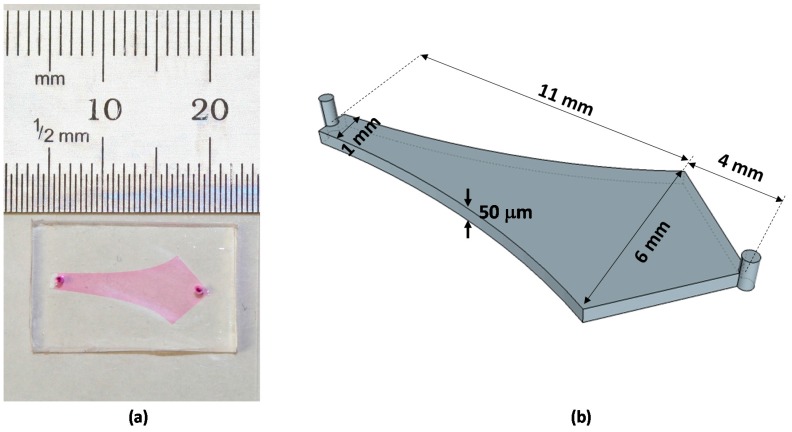
The microfluidic channel used in this study. (**a**) A photograph of the microfluidic channel; and (**b**) a schematic showing the geometry of the microchannel.

To study a combined effect of shear and surface adhesion strength on the alignment of filamentous bacteria, a Hele-Shaw device was used in this study, which allowed a continuous range of shear rates to be studied within a single device at a fixed inlet flow rate [53]. The device walls were functionalized with proteins having different mannosylation levels or left untreated to control the cell-surface adhesion strength. A photograph and schematic of the device is shown in Figure 1.

**Figure 2:**
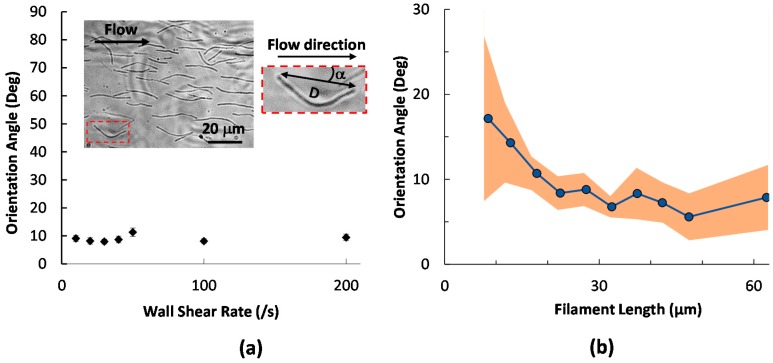
Initial alignment of adhered filamentous cells with flow. (**a**) Orientation angle as a function of wall shear rate. Each data points shows mean orientation angle with standard error measured from 75–100 filaments. The left image in the inset shows typical alignment immediately after adhesion. The filament in the red box is zoomed in and showed as the right inset. Parameters characterized in this paper include filament length, the orientation angle, i.e., the absolute value of the angle between the two end points and the flow (α), and the end-to-end distance (*D*); (**b**) Initial orientation angle as a function of filament length. The dots are averages of the absolute end-to-end angles in 10 bins as described in the text. The shaded area represents 95% confidence interval of the mean. The line connecting the dots is to guide the eye.

Under a continuous flow on all surfaces, filamentous cells mostly adhered along the flow direction. As an example, the inset of Figure 2a shows the morphology of modified fimbriated (*fim^+^*) cells captured on bare glass at a wall shear rate of ~50 s^−1^, and the flow direction is indicated by the arrow. Beyond natural curvatures, which were observed for filamentous cells in the planktonic state, the end-to-end orientation of the majority of the cells deviates little from the flow direction. The orientation angle (absolute value of the angle between the two end points and the flow direction, α in Figure 2a right inset, 0° ≤ α ≤ 90°) is around 10° under all wall shear rates tested between 10–200 s^−1^, commonly encountered in physiological environments. The value is expected to be 45° when the filaments are randomly distributed. Thus, shear stress is effective in aligning filamentous cells during their adhesion.

The drag on a thin and rigid filament in a low Reynolds number flow is anisotropic, with a greater resistance when oriented perpendicular rather than parallel to the flow. This effect, together with the shear gradient in a microchannel, aligns filament-shaped subjects with the flow direction. However, a small number of adhered cells were seen to align at large angles from the flow, due to rotation of the filamentous cells propelled by flagellated swimming. When the cells were injected into the device, a majority were observed to align with, and move along, the flow direction, while a small number were found to swim upstream or sideways. Migration of bacteria has been found to be strongly shear-dependent. For example, Kaya et al. reported that the swimming trajectory of *E. coli* changed from a circular pattern at no flow to rheotaxis at low shear. At higher shear on the order of 10 s^−1^, *E. coli* swam orthogonal to the flow and got dragged downstream by the flow [54]. Since the shear rates used here are all relatively high [55], flow alignment is the predominant phenomenon. A small number of fastest swimmers could still overcome the flow and migrate sideways or even upstream, leading to large orientation angles upon adhesion. 

We further inspected whether the orientation was influenced by the length of the filamentous cells, with wall shear rates between 10 and 200 s^−1^. The dots in Figure 2b are orientation angles averaged for filaments binned at <10 μm, >50 μm and in the range of 10–50 μm with 5-μm intervals. The shaded area represents 95% confidence interval of the average. As observed in Figure 2b, filamentous cells shorter than 15 μm in length align more randomly from the flow direction, thus having greater orientation angle averages and wider confidence intervals. As the length increases, the cells align more consistently with the flow, bringing the orientation angle to around 10°.

Interestingly, the dependence of cell orientation on filament length differs from Hill et al.’s observation using nonfilamented cells [55], where the body angle with the flow direction increased from ~65° to ~80° when the aspect ratio of *E. coli* increased from 1.5 to 5.5. Hill et al. also observed that the cell body was more perpendicular to the flow as the wall shear rate increased. The preferential transverse alignment of *E. coli* relative to the flow direction was attributed to torque generated by flagellar bundle rotation. In our study, the filament length is beyond those tested in Hill’s work. Nonetheless, larger alignment angles of short filaments seem to match the observation by Hill et al. For filaments longer than 15 μm, coordinated flagellar rotation may become more difficult and the shear effect dominates, leading to improved filament alignment with the flow. 

### 2.2. Filament Elongation on Substrates with Different Affinity

Cell adhesion is critical to surface colonization and biofilm formation [56]. Adherence is, therefore, a favorable cell state. *E. coli* use outer membrane organelles, including type 1 fimbriae, for adhesion, which promote overall adherence to hydrophobic surfaces and also mediate specific binding interactions with glycosylated surfaces containing mannose. We used cell fimbriation as a tool to elucidate a relationship between filamentous growth and surface adhesion. 

**Figure 3:**
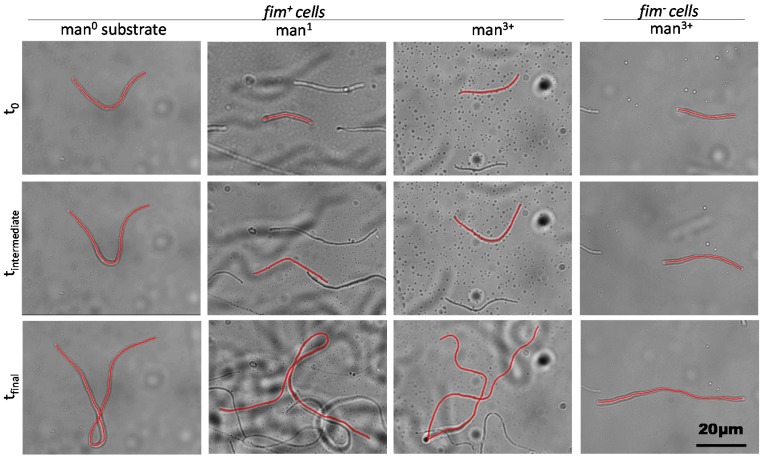
Time-course imaging of filamentous cells in microfluidic channels with varied mannosylation. Representative images show filamenting cells in the same field of view at the initial time point (t_0_), an intermediate time point (t_intermediate_, ~20 min) and after elongation ceased (t_final_). Cells were either fimbriated (*fim*^+^) or non-fimbriated (*fim*^−^) and attached to substrates with no mannosylation (man^0^), monomeric mannose (man^1^), or oligomeric mannose (man^3+^). Red lines trace single cells tracked over time and remaining in the field of view throughout the time course.

To do so, substrates were developed within the microfluidic device to promote differing levels of adhesion strength for the mannose-specific interaction. For *in vitro* studies, bovine serum albumin (BSA) conjugated with a mannose derivative is often used to functionalize surfaces with monomeric mannose by protein adsorption [57]. RNAse B, a mannosylated enzyme with oligomeric residues, is again useful to obtain a representative surface of high binding by adsorption methodology [14,58,59]. For this work, three protein-based substrates supplied varying mannosylation patterns, including BSA for a mannose-absent surface (man^0^), BSA-mannose for monomeric mannose functionalization (man^1^) or RNAse B for oligomeric mannose functionalization (man^3+^); thus, through fimbriae-mediated mannose binding, relative surface adhesion of cells corresponded to the surface mannose content. Proteins were incubated in-channel to achieve sufficient protein adsorption to the surface; equivalent monolayer coverage was verified for all proteins via quartz crystal microbalance (QCM) measurements. Then, after washing, early-stage filamentous cells, either fimbriated (*fim^+^*) or lacking fimbriae (*fim^−^*), were introduced to the channel with aztreonam and allowed to adhere under flow. Fimbriated cells bound readily to mannose-modified substrates, while nonspecific binding on man^0^ required more time to obtain the same density of cells within the field of view. Few *fim^−^* cells bound to any of the tested substrates even after an hour of cell injection. Subsequently, the floating cells were rinsed by flowing cell-free media into the channel, and time-course microscopic imaging was used to monitor filamenting cells incubated on-stage (Figure 3, Videos S1–S4 at a frame rate of 1 min per frame). Filamentation was tracked over time until growth ceased and then filament length over time was measured for cells preserved in the field of view for the entire series. To maintain the cells in the filamentation state, the cell-free media were supplemented with 10 μg/mL aztreonam, without which filaments started breaking down into fragments after ~30 min (Video S5 at a frame rate of 1 min per frame).

**Figure 4:**
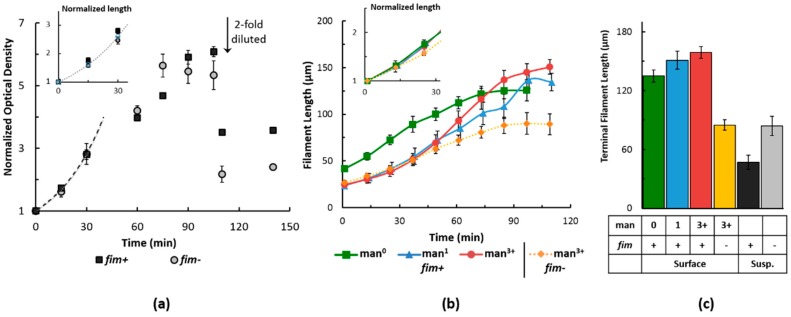
Growth trends of antibiotic-induced filamentous cells. (**a**) Comparison between nonfimbriated (*fim*^−^) and fimbriated (*fim*^+^) cells during filamentous growth in suspended cultures. Time points were measured as the optical density of the culture at 600 nm and normalized to the optical density of the same sample at 0 min. Where indicated, cultures were diluted two-fold prior to measurement and continued culturing. The inset plots early time points (x axis) as a function of filament length (measured by optical microscopy) normalized to the corresponding mean length at time zero (y axis). The dashed lines in the figure and inset show an exponential fit of the first three time points to obtain the growth rates. At least three independent samples were measured; error bars represent normalized standard error of the data; (**b**) comparison of filament growth dynamics between *fim*^−^ and *fim*^+^ cells during filamentous growth when attached in microfluidic channels with varied mannose content. The substrate was either not mannosylated (man^0^), or presented either monomeric (man^1^), or oligomeric mannose (man^3+^). Time points show mean filament length with standard error, calculated from roughly 10 cells that were analyzed in duplicate independent experiments. The inset highlights the early time points (x axis) as mean normalized lengths (y axis), calculated from the length per cell as a ratio to its initial length; and (**c**) terminal length of filamentous cells on different substrates vs. suspended culture after reaching a stationary growth phase, measured from roughly 25 cells in duplicate experiments; the error bars are standard error from the terminal length.

Additionally, filamenting cultures were monitored for growth rate in suspension (Figure 4a) and on surfaces (Figure 4b) and for terminal length after growth ceased (Figure 4c). Of note, termination of growth in suspension was confirmed by diluting cultures in half with fresh nutrient medium upon reaching a stationary phase and observing no subsequent increase in culture density (Figure 4a). The doubling times obtained for all the growth conditions are compiled in Table S1 in the Supplementary Materials. The exponential growth rates of *fim^−^* and *fim^+^* cells under filamenting conditions were identical during suspended culturing, with an estimated exponential doubling time of ~20 min, observed by a normalized growth curve, plotted as a ratio of the optical density to the initial condition at 0 min (Figure 4a). Since optical density is not fully reliable when cell shape changes, the doubling time was also checked by length measurements with optical microscopy (Figure 4a, inset) and found to be identical.

The growth of bacterial filaments on the varied surfaces is shown in Figure 4b, with doubling rates shown in the inset of Figure 4b by normalizing to the initial length. In general, in-channel growth was slower compared to suspended culture, but this behavior was more dramatic for *fim^−^* cells: their average doubling time slowed to 37 min, while *fim^+^* cells maintained an average doubling time of 30–33 min in-channel, depending on the surface. The individual growth trends of *fim^+^* cells on various substrates were not statistically different, while the slower growth rate of *fim^−^* cells relative to the *fim^+^* cells averaged across the three surfaces was of statistical significance at *p* < 0.05.

Additionally, we observed that the terminal length of the filamentous cells varied dramatically depending on the adhesion capability of the cells and substrate condition. *Fim^−^* filaments grown on a man^3+^ surface were the shortest. Their lengths were on average 89 μm and, further, they were statistically found to be in the same range when grown on a surface as when grown in suspension (Figure 4c). The *fim^+^* cells, however, differed significantly between suspension and adherent cultures. In solution, filaments were stationary at a mean length of 46 μm, which is consistent with previously reported lengths [29], yet 44% shorter than *fim^−^*. On surfaces, though, the average lengths ranged from 135 to 159 μm. Whereas the *fim^−^* filaments showed no change in terminal length due to surface binding, the *fim^+^* filaments showed enhancement at least 2.5-fold over the suspended culture terminal length. The difference in suspension and surface terminal lengths for the *fim^+^* cells is statistically significant at *p* < 0.001 and the difference between the *fim*^+^ and *fim*^−^ cells on the surfaces is statistically significant at *p* < 0.005. 

Further, the surface-bound length of *fim^+^* cells appeared to be influenced by the presumed surface interaction. Respective of the different surface chemistries, mean filament length increased from 135 μm on man°, to 151 μm on man^1^, and finally to 159 μm on man^3+^ (Figure 4c); the difference between the man^3+^ and man^0^ surfaces is statistically significant at the *p* < 0.01 level, while the difference between the man^3+^ and man^1^ surfaces is not as significant. Therefore, for the *fim*^+^ strain, filaments on man^1^ showed intermediate elongation, having some similarity in length measurements to filaments on each of the alternative surfaces. This fit within a more general outcome of distinct contrast between filamentation on a mannose-absent surface (man^0^) and a surface rich in oligomeric mannose (man^3+^).

These results have shown a trend between the cell-to-surface adhesion potential and the filamenting length. Nonfimbriated cells do not adhere well to the substrate, shown with the man^3+^ surface, and exhibit the slowest growth, ultimately reaching shorter lengths. Fimbriated cells, however, grow faster and remarkably longer than nonfimbriated cells in contrast to their behavior in suspended culture. Moreover, the results suggest that the binding strength of the interaction play a role in further enhancing the filament length. A man^0^ surface should have only nonspecific contributions to cell binding, while man^1^ and man^3+^ enable specific interaction, with man^3+^ promoting the tightest fimbrial binding conformation [60]. Correspondingly, the terminal filament length was further elongated on mannosylated surfaces, exhibiting a minor shift on man^1^, but a readily distinguishable outcome on man^3+^. Type 1 fimbriae have been shown to be fundamental to *E. coli* attachment. It has been reported that the specificity of the fimbrial tip to bind mannose enables initial contact [61]. The fimbrial rod as a whole establishes an alternative or secondary, nonspecific contact, especially to hydrophobic surfaces [62], and may even shorten upon attachment to bring the cell closer to the surface [63]. Thus, fimbriated cells maintain superior attachment compared to nonfimbriated cells in a manner that is independent of, but further enhanced by, the presence of mannose. Subsequently, surface attachment is known to induce changes in the outer membrane protein composition and also introduces membrane perturbations that lead to an upregulation in the periplasmic stress response system of *E. coli* as a corrective measure [61,64,65].

Aztreonam-induced filamentation occurs with an absence of regulatory changes to gene expression, especially because its mechanism of action precludes an SOS response [29]; it may, nevertheless, make the cells susceptible to crosslinking defects at the septal ring that lead to membrane bulging and eventual lysis [28]. The loss in membrane potential of filamentous cells over time has been reported and is an indicator of the decline in viability of filaments [66]. On the other hand, attachment could indeed influence both the cellular and physical response that prolongs cell viability. Thus, we expect that the propensity and strength of cell attachment may promote adaptive characteristics to otherwise fragile filaments and enhance their elongation potential.

Overall, these observations have shown a favorable effect of cell adhesion on filament elongation. Filamentous *E. coli* on uroepithelial cells in vivo have been previously reported to reach 80 μm in length and, presumably, use mannose contacts for strong adhesion [5,24]. Aztreonam-induced filaments, however, have only been reported to reach a maximum of 43 μm in length before lysing, except when surface-bound [29,67]. Thus, the glycoprotein-laden adhesive surface provided to filamenting *E. coli* in this work may offer a tissue-like substrate to facilitate filamenting behavior that is closely representative of natural mechanisms of *E. coli* persistence.

### 2.3. Dynamic Geometry of Filamentous Bacteria during Elongation 

The strength of interactions between the growing filaments and the surface influences the shape the filaments take in addition to affecting the filament terminal length. Qualitatively, this can be seen in the microscope images in Figure 3 and Videos S1–S4, where the *fim^+^* filaments buckle and form loops while the *fim^−^* filaments remain straight and aligned with the flow direction. The buckling arises during the growth of *fim^+^* filaments on the surface from the strong interactions between the fimbriae and the surface. The growing cell wall tends to extend the filament along its axis but this requires the ends of the filament to slip on the surface. When the force adhering the filament to the surface is very weak (as is the case for the *fim^−^* filaments), the ends readily extend, but when the adhesion force is stronger, there is a competition between the adhesion force and the force exerted by the growing cell wall; when the adhesion force prevents the ends of the cells from extending, the growing filament buckles instead [68,69,70]. This buckling is a general phenomenon observed whenever a force acts to compress a filament and can be induced in bacterial filaments not only from surface interactions but also by other mechanisms, such as osmotic stress or reinitiation of cell division [71].

**Figure 5:**
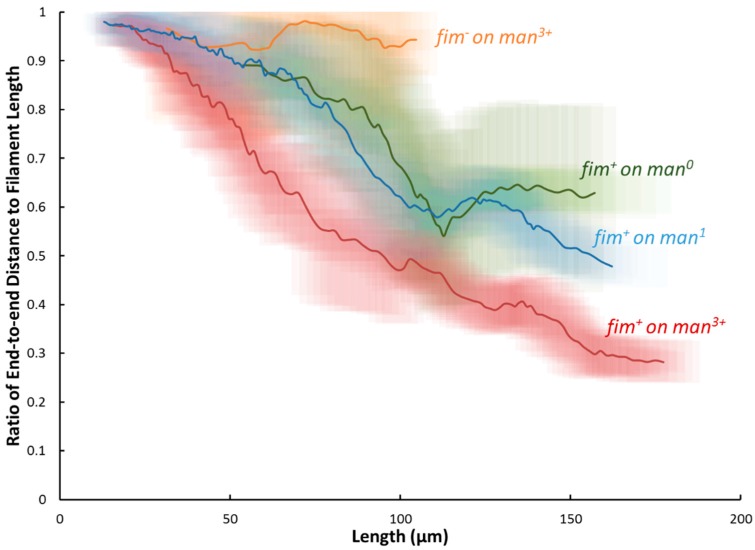
The ratio of the end-to-end distance to total length plotted as a function of the total length. The lines show moving averages and the shaded region around each line shows the region encompassed by one standard deviation away from the average. The lines are labeled *fim^−^* for filaments without fimbriae (grown on the man^3+^ surface) and with different surfaces compositions for *fim^+^* filaments. The *fim^−^* filamentous bacteria grow nearly fully extended resulting in ratios nearly one for all lengths. The *fim^+^* bacteria buckle rather than grow fully extended resulting in lower ratios, with the extent of buckling increasing with the extent of surface mannosylation.

To quantify the extent of buckling, the distance between the ends of the filaments was divided by the total length of the filament. If the filament is fully extended, the end-to-end distance is equal to the total length of the filament and the ratio is equal to 1. As the filament buckles and becomes more coiled the ratio decreases. In Figure 5, the ratio of the end-to-end distance to total length is plotted as a function of filament total length. The lines show moving averages for *fim^+^* filaments on the three surfaces examined and for *fim^−^* filaments on the man^3+^ surface. The shaded region around each line shows the region encompassed by one standard deviation away from the average. For all the conditions examined, at short lengths the ratio is close to unity. In part, this is because the filaments tend to be fully extended in solution before they are put on the surface. (Typical ratios in solution are 0.9–0.95). Additionally, for short filaments, a smaller length needs to slip along the surface to avoid buckling, making it easier for the filaments to grow extended. As the filaments grow, there tends to be a length beyond which the filaments buckle rather than continue to extend. This length depends on the strain examined and on the degree of surface mannosylation. The *fim^−^* filaments on man^3+^ remain nearly straight for all lengths, with an average ratio of 0.95. In contrast, *fim^+^* bacteria on the man^3+^ surface begin buckling as soon as they adhere to the surface, with no minimum length for the onset of buckling. Although this onset was delayed until longer filamentation for *fim*^+^ bacteria on man^0^ and man^1^ substrates, their filamentation also showed a steady trend in buckling with length-dependent ratios comparatively similar to each other. Overall, between the *fim^+^* cells on any substrate and *fim^−^*, the end-to-end ratios became distinguishable (at *p* < 0.01) by the 75 μm elongation point. Further, the ratios of all *fim*^+^ cells continued to decline with elongation, diverging in a substrate-dependent manner (at *p* < 0.01) for filaments greater than 145 µm; resultantly, the end-to-end spread of the longest comparable filaments (at ~ 157 µm) was statistically different (at *p* < 0.01) across the various substrates at a ratio of 0.63 on man^0^, 0.50 on man^1^, and 0.30 on man^3+^. From these trends, the presence of fimbriae-mediated adhesion leads to a buckling phenomenon during filamentation on surfaces, where the length ratio is a metric of buckling. Buckling during filamentation was initiated most readily on the man^3+^ substrate and, above a threshold length of 145 µm, resulted in an inverse relationship between the length ratio and mannose content.

The trends with buckling can be compared with the growth kinetics and terminal length (Figure 4). In particular, more buckling is correlated with longer terminal length and this buckling, when strongly bound to the surface, limits the distances the two ends of the filaments can span. Even though some of the *fim^+^* filaments reach lengths over 150 μm on the man^3+^ surface, the ends of filaments are never further than 60 μm apart. In contrast, the weakly bound *fim^−^* filaments often have ends separated by 90 μm, similar to their total length. As shown in Figure S1, in all cases, the ends of the filaments remain roughly aligned with their initial direction; initially the filaments are aligned within 10° of the flow direction and the end points remain aligned within 20° of the flow direction, even in cases where extensive buckling was observed (e.g., *fim^+^* on the man^3+^ surface). Within the shear rate tested in this study, no observable difference of the terminal length or buckling behavior was noticed with each strain on a selected surface (Figure S2). We speculate that the shear rates used in this study may be too low to have a significant effect but that higher shear rates/stresses might be strong enough to alter the surfaces effects that we have observed. Overall these results suggest that the optimal filament-surface adhesion depends on the applications, with strong adhesion promoting longer filament lengths and faster growth kinetics, while weaker adhesion reduces the degree of buckling.

## 3. Materials and Methods 

### 3.1. Cell Culturing and Filamentation

The strain AAEC191A (MG1655 r*ecA*^−^, *fim*^−^) + pPKL114 (*fim* operon ∆*fimH*, Amp^R^) and pGB (*fimH-j96*, Cm^R^) were generously donated from E. V. Sokurenko [14,59,72]. The strain AAEC072 (also MG1655 *fim*^−^) was donated from I. C. Blomfield [73]. Fimbriae-expressing (*fim^+^*) *E. coli* cells used AAEC191A transformed with pPKL114 and pGB, which supplied the genes for the fimbriae assembly apparatus and the tip adhesion (FimH) with constitutive expression. The *fim^−^* strain used was AAEC072, which lacked the entire fimbrial organelle. The modified *fim^+^* strain was AAEC191A + pPKL114 + pGB-I52-His6Au2 (a pGB derivative) [74]. All strains were cultured in LB-Miller broth (ThermoFisher, Nazareth, PA, USA) with appropriate antibiotics where necessary (100 µg/mL ampicillin and 38 µg/mL chloramphenicol) at 37 °C and 225 rpm shaking.

For filamentation experiments, growth media were inoculated with 2% of overnight cultures. When the fresh cultures emerged from the lag phase, the filamentation-inducing antibiotic, aztreonam (Sigma Aldrich, St. Louis, MO, USA), was supplemented at 10 μg/mL with brief culturing conditions to promote a “prefilamentation” state before injection into a microfluidic flow channel. Alternatively, cells were cultured in suspension with aztreonam for 2.5 h. Growth rates during shaking conditions were monitored by measuring the optical density of biological triplicates using a UV-VIS spectrophotometer at 600 nm. In parallel, cell samples taken from culture were imaged at corresponding time points to measure the filament length of at least five cells per frame.

### 3.2. Device Fabrication 

Polydimethylsiloxane (PDMS) microchannels (Figure 1) were prepared following the standard soft lithography protocol. Briefly, SU8 of 50 μm thickness was patterned on silicon wafers by photolithography and was used as the mold. A 10:1 mixture of silicone elastomer base and silicone elastomer curing agent (Sylgard 184 silicone elastomer kit, Dow Corning Corporation, Midland, MI, USA) was poured onto the mold, degassed, cured at 65–75 °C, and the microdevices were cut out. Fluid inlets and outlets were drilled using a syringe needle. Microchannels and glass coverslips were then activated by oxygen plasma and the exposed surfaces were brought into contact. The assembled devices were heated for 5–10 min at 65–75 °C to produce permanent bonding.

### 3.3. Surface Modification

The microchannel walls were coated with one of three proteins: bovine serum albumin (BSA), mannosylated BSA, or RNase B, by physisorption. Mannose-derivatized BSA (Vector Laboratories, Burlingame, CA, USA) was purchased as a synthetically-conjugated neoglycoprotein. RNAse B (New England Biolabs, Ipswich, MA, USA) is naturally glycosylated with a variety of mannose-rich heptasaccharide structures that expose at least an oligomannose-3 chain. As a result, each protein molecule introduces 0, single and ≥3 mannose, and are noted as man^0^, man^1^, and man^3+^ in the manuscript. The proteins were dissolved in 2:1 methanol/water mixture at a final concentration of 250 μg/mL. Five microliters of solution were injected into the microfluidic channel through the inlet port using a pipette tip and the device was left at room temperature for one hour to dry out. Afterwards, the channels were rinsed with copious amount of water and used immediately.

The adsorption of proteins onto surface from solution was examined with a quartz crystal microbalance (Novaetech openQCM, Naples, IT) with a 10 MHz resonant frequency and mass sensitivity of 4.42 ng/(cm^2^ Hz). For all three proteins a frequency shift of approximately 150 Hz was observed over 10–15 min when the solution was placed on the QCM crystal; no such shift was observed when only methanol–water mixtures were used. This corresponds to a mass shift of 6.6 ng/cm^2^. Using a density of 1.3 g/cm^3^ (the density of BSA), this mass shift indicates a monolayer of protein with 5 nm thickness, reasonable estimates for both BSA and RNase B (BSA is approximately 14 nm × 4 nm × 4 nm, and RNase B is 10 nm × 3 nm × 7 nm). Such coverage should expose many more mannose groups than FimH groups displayed by a bacterium. Specifically, given the dimensions of the proteins, there should be greater the 10,000 protein molecules per square micron versus a few hundred fimbriae per bacterium.

### 3.4. Experimental Setup for Flow Experiments

Capture and culture of filamentous cells on microfluidic chips were performed on the stage of an inverted microscope (Nikon Eclipse TE2000, Melville, NY, USA). Bacterium cultures prefilamented for 30–45 min at 37 °C in a shaking incubator were injected into the devices using a syringe pump. After a satisfactory number of filamentous cells (defined below) were immobilized on the surface, the culture suspension was replaced with cell-free LB-miller medium supplemented with 10 μg/mL aztreonam continuously flowing at the same rate. To inspect initial cell alignment under different wall shear rate, the culture suspension was injected at either 1 or 5 μL/min and replaced with cell-free media after randomly-selected image frames of 150 μm × 200 μm had ~100 immobilized filamentous cells. Afterwards, the device floor was imaged using a 40× objective at 3–4 locations at the middle line along the flow direction. The geometry of the flow channel combined with two flow rates allows the study of a continuous range of wall shear rates between 10 s^−1^ to 200 s^−1^ [75]. The wall shear rate was calculated using $\dot{\gamma }=6Q/(wh^{2})$, assuming a laminar flow, no slip boundary condition, and negligible edge effect from the side walls. $\dot{\gamma ,}Q,w,\;\text{and h}$ are the wall shear rate, volumetric flow rate, channel width, and height, respectively.

For the on-chip cultures, the culture suspension was injected at 5 μL/min and replaced with the cell-free media after ~5–10 cells were seen to immobilize in an image frame located in the center of the device, corresponding to a wall shear rate of ~50 s^−1^. This cell density was selected to avoid significant cell overlapping after their length extended to 100 μm or more. It should be noted that depending on the cell strain and surface chemistry, the speed of cell immobilization varied significantly: on man^3+^ and man^1^ surfaces, *fim^+^* cells immobilized quickly and sometimes reached 5–10 cells per frame within minutes. After switching to the cell-free media, floating cells upstream of the imaging frame continued to capture during the rinse, leading to more than expected cells. The *fim^+^* cells required ~30 min to reach ~5 cells per frame on the man^0^ surface, with little additional binding after switching to a cell-free medium. On the other hand, surface-bound *fim^−^* cells was always sparse and rarely reached more than three cells per frame even after an hour of cell injection. To capture early stage cell growth on chip, *fim^−^* cells were injected for 30 min before switching to cell-free media. 

A home-built stage warmer was then used to maintain the microfluidic chip at 37 °C and time-lapsed images were acquired every minute for 2 h from the same location near the device center along the flow direction, where the wall shear rate was about 100 s^−1^. 

### 3.5. Image Analysis

In every thirteenth image, the filamentous bacteria were traced using the NeuronJ plugin for ImageJ (National Institutes of Health, Bethesda, MD, USA). Traces for any bacteria that ultimately grew out of the frame were discarded. The traces produced by NeuronJ were imported into Microsoft Excel for analysis to obtain the filament length, the orientation angle, the end-to-end distance, and its ratio with the filament length. The end-to-end distance is simply the distance between the two end points of the bacteria, while the orientation angle is the absolute value of the angle formed by the two end points with the flow direction. When determining the role of length on the buckling of the bacteria (Figure 5), images taken in the on-chip cultures after bacterial growth had ended were combined with data from the videos; this ensured a large number of data points (typically 100) for each line. Each line represents a 10-point moving average with the shaded area representing one standard deviation away from the line.

## 4. Conclusions 

Here, we examined the behavior of filamentous bacteria on and near surfaces while under shear. When under shear, bacterial filaments longer than 15 μm adhere to a surface aligned with the flow direction, initially. The growth of the filamentous bacteria on the surfaces was shown to strongly depend on the bacteria’s affinity for the surface as mediated by type 1 fimbriae. Specifically, for *fim^−^* bacteria, where fimbriae were not expressed, the bacterial filaments grew in a similar manner to their growth in solution. The terminal length the bacteria reached was similar in solution and on the surface, and in both cases, the bacterial filaments grew in an extended manner, with minimal curvature. In contrast, *fim*^+^ bacteria grew to a much longer terminal length on the surface than in solution. Additionally, bacterial filaments on the surface buckled into coiled and looped shapes, rather than remaining fully extended. Surfaces with different extents of mannosylation were examined with the *fim^+^* bacteria, since the *E. coli* type 1 fimbriae is known to strongly attach to mannosylated proteins with the affinity varying with the degree of mannosylation. It was found that the strongest binding interactions due to oligomeric mannose led to increased terminal length of filamentous bacteria and contributed to earlier onset buckling behavior, while the extent of mannosylation resulted in a more confined terminal geometry among the longest cells. From these results, it is clear that the growth and geometry of filamentous bacteria on surfaces is modulated by their affinity for the surface. Since many surface-attached pathogenic bacteria naturally form filaments when stressed, the role of surface affinity in the behavior of filamentous bacteria has many biomedical implications. Additionally there are important implications for using filamentous bacteria as templates for inorganic structures, as increasing the bacterial affinity for the surface may promote the formation of longer structures, at the expense of their straightness.

## Supplementary Materials

Supplementary materials can be accessed at: http://www.mdpi.com/1420-3049/21/8/985/s1.
