# Supplementary material for: Influences of Adhesion Variability on the “Living” Dynamics of Filamentous Bacteria in Microfluidic Channels
Source: Molecules. 2016 Jul 28;21(8):985. doi: 10.3390/molecules21080985 (PMC6274349; doi:10.3390/molecules21080985)
Supplement: Supplementary file 1 [file molecules-21-00985-s001.zip › molecules-134620-supplementary/Supplementary Tables and Figures.pdf]

Supplementary Materials: Influences of Adhesion Variability on the "Living" Dynamics of Filamentous Bacteria in Microfluidic Channels

Justin P. Jahnke, Jessica L. Terrell, Austin M. Smith, Xuanhong Cheng and Dimitra N. Stratis-Cullum

Table S1. Doubling time for filamenting *fim*$^+$ and *fim*$^-$ cells in suspension and on the different surfaces. Error represents a 95% confidence interval.

On surface			Doubling time (min)
	fim$^-$	man^{3+}	37 ± 4
	Fim$^+$	man^0	30 ± 3
		man^1	30 ± 3
		man^{3+}	33 ± 5
in suspension			
• OD			
	fim$^-$		21 ± 6
	fim$^+$		20 ± 3
• microscope			
	fim$^-$		20 ± 3
	fim$^+$		23 ± 5

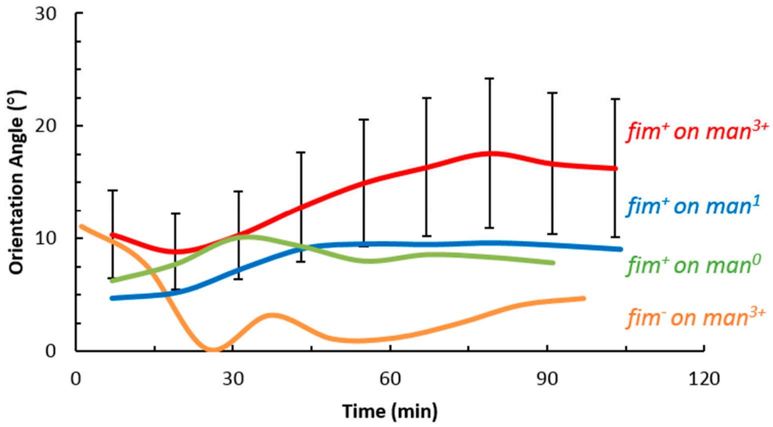

Figure S1. Dynamic orientation angle during cell filamentation on substrates. The orientation angle between the ends of the growing filaments and the flow direction is shown as a function of time for the various surfaces and bacterial strains examined. Error bars representing the standard error are shown for *fim*$^+$ on man^{3+}; the error bars for the other lines are omitted for clarity but are of similar sizes or smaller than *fim*$^+$ on man^{3+}.

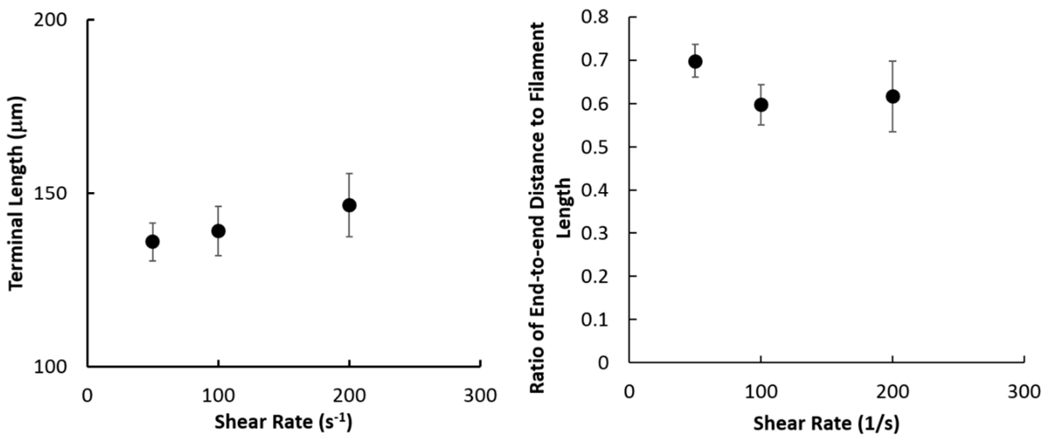

Figure S2. Investigation of shear effect on terminal length and buckling geometry of *fim*+ filamentous cells. Terminal length (left) and ratio between end-to-end and terminal lengths (right) for *fim*+ filaments on BSA substrates as functions of shear rate. The error bars represent standard error from measurements of at least 8 filaments.
